# Detecting and discriminating novel objects: The impact of perirhinal cortex disconnection on hippocampal activity patterns

**DOI:** 10.1002/hipo.22615

**Published:** 2016-07-22

**Authors:** Lisa Kinnavane, Eman Amin, Cristian M. Olarte‐Sánchez, John P. Aggleton

**Affiliations:** ^1^ School of PsychologyCardiff University70 Park Place, CardiffWalesCF10 3ATUnited Kingdom; ^2^ Present address: Cristian M. Olarte‐Sánchez is currently at Rowett Institute of Nutrition and Health, Institute of Medical Sciences, Aberdeen University, ForesterhillAberdeenAB25 2ZDUK

**Keywords:** entorhinal cortex, hippocampus, nucleus reuniens, prefrontal cortex, recognition memory

## Abstract

Perirhinal cortex provides object‐based information and novelty/familiarity information for the hippocampus. The necessity of these inputs was tested by comparing hippocampal c‐*fos* expression in rats with or without perirhinal lesions. These rats either discriminated novel from familiar objects (Novel‐Familiar) or explored pairs of novel objects (Novel‐Novel). Despite impairing Novel‐Familiar discriminations, the perirhinal lesions did not affect novelty detection, as measured by overall object exploration levels (Novel‐Novel condition). The perirhinal lesions also largely spared a characteristic network of linked c‐*fos* expression associated with novel stimuli (entorhinal cortex→CA3→distal CA1→proximal subiculum). The findings show: I) that perirhinal lesions preserve behavioral sensitivity to novelty, whilst still impairing the spontaneous ability to discriminate novel from familiar objects, II) that the distinctive patterns of hippocampal c‐*fos* activity promoted by novel stimuli do not require perirhinal inputs, III) that entorhinal Fos counts (layers II and III) increase for novelty discriminations, IV) that hippocampal c‐*fos* networks reflect proximal‐distal connectivity differences, and V) that discriminating novelty creates different pathway interactions from merely detecting novelty, pointing to top‐down effects that help guide object selection. © 2016 The Authors Hippocampus Published by Wiley Periodicals, Inc.

## INTRODUCTION

Models of medial temporal lobe function distinguish two pathways that converge upon the hippocampus, one for “what (item)” information, the other for “where (context)” information (Eichenbaum et al., 2007; Ranganath and Ritchey, 2012). The perirhinal cortex occupies a central position in the “what” pathway as it conveys high‐resolution object information to the entorhinal cortex and hippocampus (Fernandez and Tendolkar, 2006; Bussey and Saksida, 2007; Deshmukh et al., 2012; Suzuki and Naya, 2014), while also signaling the novelty/familiarity of this same information (Brown and Aggleton, 2001; Ho et al., 2015). Evidence for this “what” pathway includes findings from rat lesion studies, which show how perirhinal cortex damage disrupts object recognition (Brown and Aggleton, 2001; Winters et al., 2008), alongside evidence for the joint involvement of the perirhinal cortex with the hippocampus for associative object recognition tasks, such as discriminating a familiar object set in a novel spatial location (Barker and Warburton, 2011a, 2011b; Warburton and Brown, 2015). In addition, functional imaging studies reveal that both the hippocampus and perirhinal cortex are active in humans engaged in similar object‐position related tasks (Diana et al., 2007; Hsieh et al., 2014). Despite these findings, both behavioral and clinical studies indicate that, following the loss of perirhinal cortex, the hippocampus can sometimes still support complex visual discriminations by rats and episodic memory in humans (Graham and Hodges, 1997; Winters et al., 2004; Bowles et al., 2007, 2010, 2011; Aggleton et al., 2010).

To understand these seemingly conflicting results, the present experiments examined whether perirhinal integrity is required for the hippocampal c‐*fos* activity associated with processing novel item information. The immediate‐early gene (IEG) c‐*fos* provides an indirect signal of neural activity (Tischmeyer and Grimm, 1999; Guzowski et al., 2005), with perirhinal expression increasing after exposure to novel visual stimuli (Wan et al., 1999; Kinnavane et al., 2015). This increase is associated with characteristic patterns of interlinked c‐*fos* activity across medial temporal lobe sites (Albasser et al., 2010; Kinnavane et al., 2015; Mendez et al., 2015). Furthermore, c‐*fos* expression stabilizes long‐term recognition memory in perirhinal cortex (Seoane et al., 2012) while its production can be used to record and reactivate contextual learning within the hippocampus (Liu et al., 2012; Ramirez et al., 2013). Thus, c‐*fos* is an integral component of effective memory processes in medial temporal sites.

**Figure 1:**
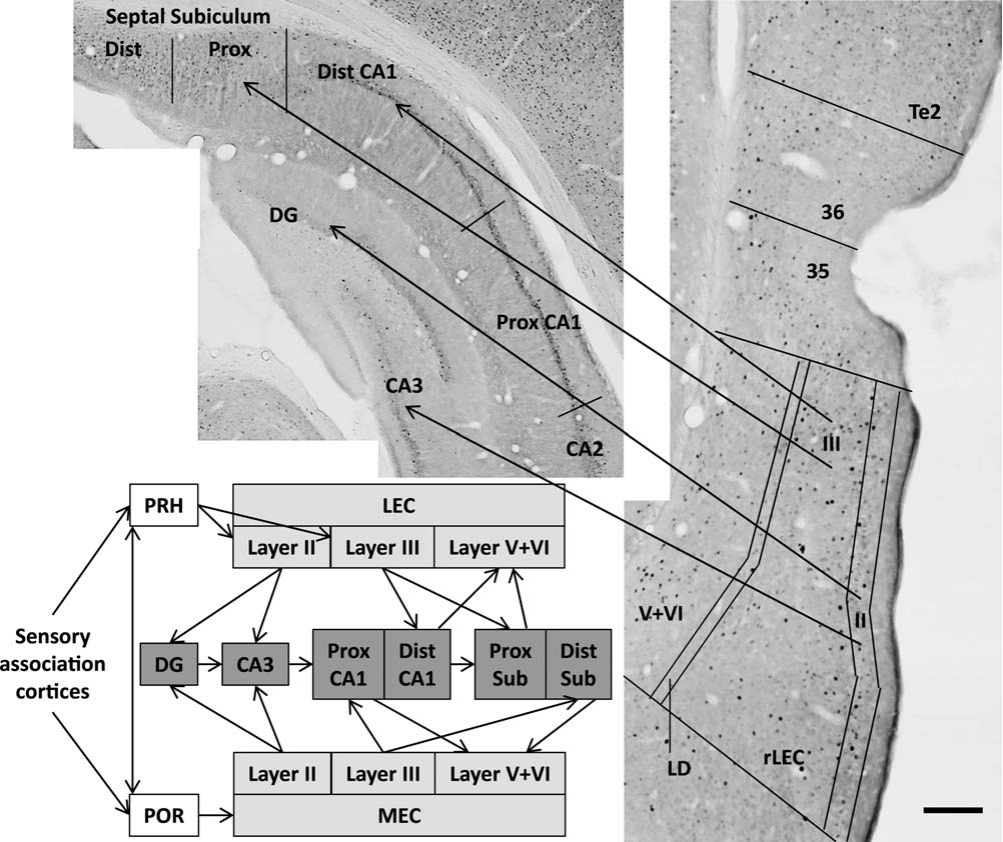
Simplified depiction of afferent inputs from the parahippocampal region to the hippocampal formation. The photomicrographs show coronal sections stained for Fos‐positive cells from a rat in the Sham Novel‐Familiar group. The regions shown on the photomicrographs include area Te2 as well as areas 35 and 36, which comprise the perirhinal cortex (PRH), and the rostral lateral entorhinal cortex (rLEC) with cortical layers (II, III, V, + VI) delineated. For simplicity, the schematic does not include the direct connections linking the perirhinal cortex and the postrhinal cortex (POR) with CA1/subiculum, or the direct connections between the LEC and medial entorhinal cortex (MEC). Other abbreviations: Dist, distal; DG, dentate gyrus; LD, lamina dessicans; Prox, proximal. Scale bar 200 μm.

Previous c‐*fos* studies have repeatedly revealed two, distinctive entorhinal pathways. Discriminating novel from familiar objects preferentially engages entorhinal projections to the dentate gyrus/CA3, via the perforant pathway. In contrast, familiar objects preferentially engage the direct entorhinal projections to CA1, via the temporoammonic pathway (Albasser et al., 2010; Kinnavane et al., 2014; Olarte‐Sanchez et al., 2014). The latter pathway has also been associated with the maintenance of familiar spatial memories (Remondes and Schuman, 2004; Poirier et al., 2008). If perirhinal signals of novelty trigger this pathway difference then, in the presence of novel objects, perirhinal lesions should bias c‐*fos* activity away from the “novel” entorhinal→dentate gyrus/CA3 pathway to the “familiar” entorhinal→CA1 pathway. This same bias should cause rats with perirhinal cortex lesions to behaviorally treat novel stimuli as familiar (McTighe et al., 2010; Romberg et al., 2010), i.e., reduce the exploration of novel objects. Consequently, one objective was to contrast activity in entorhinal Layer II, which projects to the dentate gyrus and CA3, with entorhinal Layer III, which projects to CA1 (Steward and Scoville, 1976; Fig. 1). Based on their segregated connectivity and the forgoing Fos results, novel objects would be expected to preferentially engage entorhinal Layer II in intact rats.

A further goal was to distinguish proximal from distal areas within both septal and temporal hippocampal subfields (Van Strien et al., 2009). This goal reflects emerging evidence from anatomical, electrophysiological, and IEG imaging studies, of distinct, functional networks involving this axis. While spatial information is thought to be preferentially processed in proximal CA1 and distal subiculum, nonspatial/object related information may be preferentially processed in distal CA1 and proximal subiculum (Witter et al., 2000; Henriksen et al., 2010; Aggleton, 2012; Schmidt et al., 2012; Hartzell et al., 2013; Nakamura et al., 2013; Knierim et al., 2014). Next, these interactive medial temporal networks were expanded to include the prelimbic cortex and nucleus reuniens of the thalamus (Prasad and Chudasama, 2013; Xu and Sudhof, 2013). This expansion was prompted by proposals that hippocampal‐prefrontal interactions are required for associative recognition (Barker and Warburton, 2011a) as they help guide patterns of object exploration based on other acquired information, such as prior contextual learning (Preston and Eichenbaum, 2013).

**Figure 2:**
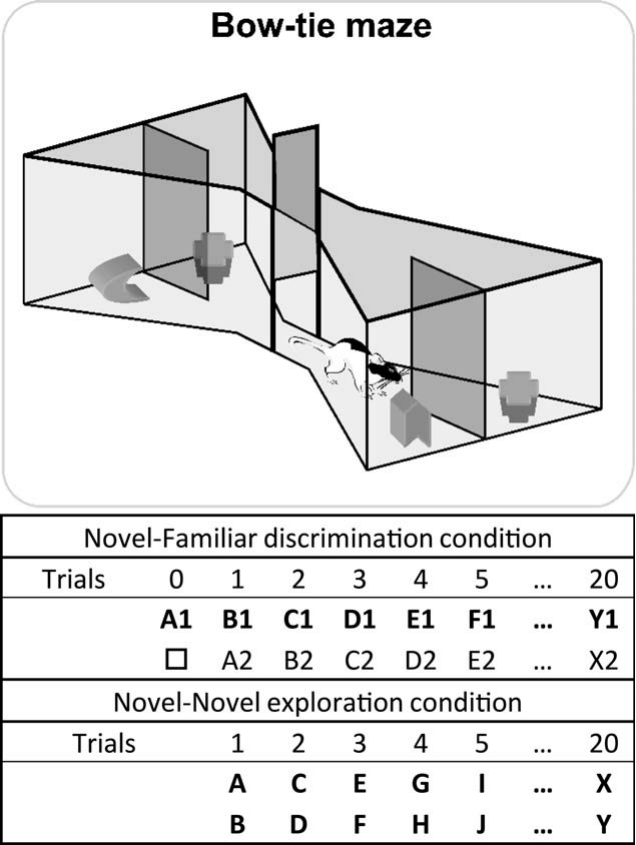
Schematic of the bow‐tie maze (upper). A central sliding door separates the two ends of the maze in which objects are placed. The rat runs back and forth from end to end of the maze, with a new trial at each end (adapted from Albasser et al., 2011a). The sequence of object pairs in the Novel‐Familiar discrimination condition (upper) and Novel‐Novel object exploration (lower) is depicted. Different objects are represented by different letters while the subscript numbers show how duplicate objects were used for the recognition trials. For the first presentation of an object, that is, when novel, the letter is in bold. The □ symbol depicts the familiar object in Trial 0 that had also been used in pre‐training. The left/right placement of novel objects was counterbalanced in the novel‐familiar discrimination condition.

To promote the expression of c‐*fos*, two behavioral conditions were employed (see Albasser et al., 2010; Olarte‐Sanchez et al., 2014), both of which contained 20 trials in a single session. The first, a Novel object‐Familiar object discrimination, consisted of multiple recognition memory trials (Fig. 2). The second, a Novel object‐Novel object pairing, allowed rats to explore freely between two different novel objects, with new objects on every trial (Fig. 2). Both conditions ensured repeated exposure to novel stimuli, but the Novel‐Novel condition removed all familiar objects as well as eliminating the spontaneous preferential selection of novel items. Consequently, it should be possible to determine if the perforant pathway recruitment of the dentate gyrus and CA3 (Albasser et al., 2010; Kinnavane et al., 2014) is merely due to the presence of novel objects or is generated by the discrimination of novel from familiar.

## MATERIALS AND METHODS

### Animals

All experiments involved male Lister Hooded rats (*Rattus norvegicus*) supplied by Harlan (Bicester, UK). The rats were housed in pairs, with water provided *ad libitum* throughout the experiment. All experiments were in accordance with the UK Animals (Scientific Procedures) Act (1986) and associated guidelines, as well as EU directive 2010/63/EU. The study was also approved by local ethical review committees at Cardiff University. Rats were kept on a 12h:12h light to dark cycle. During behavioral testing, rats were food restricted, remaining close to 85% of their free feeding body weight. Rats were approximately 9 months (Cohort A) and 8 months (Cohort B) post‐surgery at the time of c‐*fos* imaging.

### Surgical Procedures

Cohort A consisted of two groups; perirhinal cortex lesions (*n* = 17) and surgical sham controls (*n* = 12). Likewise, Cohort B contained rats with perirhinal cortex lesions (*n* = 18) and surgical sham controls (*n* = 13). The rats from both cohorts weighed between 290 g and 350 g at the time of surgery; they were around 3 months old. The surgeries for both cohorts were identical.

All rats were anesthetized throughout the surgery with isofluorane (5% for induction, 2% thereafter). The rats were placed in a stereotaxic frame (David Kopf Instruments, Tujunga, CA), with the incisor bar set at +5.0 mm to the horizontal plane. A dorsal craniotomy was made directly above the target region and the dura cut to expose the cortex. Perirhinal lesions were made by injecting a solution of 0.09M *N*‐methyl‐d‐aspartic acid (NMDA; Sigma, Poole, U.K.) dissolved in phosphate‐buffered saline (pH 7.4) in three sites in both hemispheres using a 26 gauge, 1‐µl Hamilton syringe (Bonaduz, Switzerland). The volume of NMDA injected was 0.22 µl for the rostral injections and 0.20 µl for the middle and caudal injections. The injection coordinates relative to bregma (in mm) were (1) AP −1.8, ML ±5.9, DV −9.3; (2) AP −3.4, ML ±6.2, DV −9.5; (3) AP −5.0, ML ±6.3, DV −8.9. The surgical control groups received identical treatment, except that the dura was repeatedly perforated with the same Hamilton syringe but no fluid was infused into the brain (see Albasser et al., 2015).

### Apparatus

Behavioral testing took place in a maze shaped like a bow‐tie, made with steel walls and a wooden floor (Fig. 2). The maze was 1.2 m long, 0.5 m at its widest, and 0.5 m tall. Each end of the maze was triangular in shape with the apices joined by a 0.12 m corridor. In the middle of the corridor was an opaque sliding door that divided the maze in half. Recessed into the floor, by the back wall of each triangular area, were two food wells, 3.5 cm in diameter and 2 cm deep. These wells were partially separated by a steel wall that projected 15 cm into the maze from the center of the back wall.

### Objects

Both experiments used three‐dimensional “junk” objects. Cohort A received 20 different objects, each with an identical duplicate, while Cohort B received 40 different objects. Objects differed in their color, shape, size, and texture. Any object with an obvious scent was excluded. All objects were large enough to cover a food well but light enough to be moved by a rat. The objects were cleaned with 70% alcohol wipes after each session.

### Behavioral Testing

#### Pre‐training

This phase lasted seven days. By its completion, all rats would run from one side of the maze to the other and displace an object covering a food well in order to reach a food reward (sucrose pellets 45 mg; Noyes Purified Rodent Diet, Lancaster, NH). Details of this pre‐training procedure have been fully described (Olarte‐Sanchez et al., 2014). While both cohorts then completed several object exploration tests in the bow‐tie maze (Albasser et al., 2015; Olarte‐Sanchez et al., 2015), neither cohort had been tested in the bow‐tie maze for at least 3 months prior to the present study and objects presented in current tests had not been previously used. A single rehabituation session, which involved retrieving food rewards under repeated, familiar objects at opposite ends the maze, was given two days before the test session proper. Present testing for Cohort A began 9 months post‐surgery while Cohort B were tested 8 months after surgery. This period helped to confirm that they had stable deficits on tests of object recognition.

#### Novel‐Familiar object discrimination (cohort A)

Animals were first placed in a quiet, dark room for 30 min. The subsequent behavioral test to induce c‐*fos* expression consisted of a single session of 20 continuous trials in the bow‐tie maze, each trial lasting 1 min. A single 45 mg sucrose pellet was placed in each food well, i.e., one under every object. This food encouraged engagement with the objects and ensured all rats completed the trials in a comparable amount of time (i.e., that to start each trial, they shuttled across the maze without delay). Thus, the rewards were not differentially associated with selecting the novel from the familiar object. At the start of the session the rat was placed on one side of the maze which contained a novel object (Object A) and one familiar object from pre‐training (object □; Trial 0; see Fig. 2). The rat displaced the objects to retrieve the sucrose pellet. After 1 min, the experimenter opened the door in the middle of the apparatus and the rat ran to the other side of the maze to begin Trial 1, where an identical copy of the now familiar Object A was presented alongside novel Object B (see Fig. 2). The rat could freely explore these objects for 1 min. The experimenter then opened the central door so that the rat would run to the other side of the maze to begin Trial 2, where a copy of the now familiar Object B sat alongside novel Object C, each covering a baited food well (Fig. 2). Trial 3 consisted of familiar Object C and novel Object D. This running recognition protocol continued with pairs of objects (one novel, one familiar), covering the baited food wells, until 20 trials were completed. Placement of the novel object on the left or right was counterbalanced. Immediately after testing, the experimenter returned the rat to the dark room for 90 min.

#### Novel‐Novel object exploration test (cohort B)

Once again, rats were placed in a quiet, dark room for 30 min before testing began and for 90 min at the completion of testing. The single test session consisted of 20 trials, each of 1 min. In this condition, rats explored two different novel objects on every trial (see Fig. 2). As described above, every object covered a food well baited with a sucrose pellet.

### Analysis of Behavior

**Figure 3:**
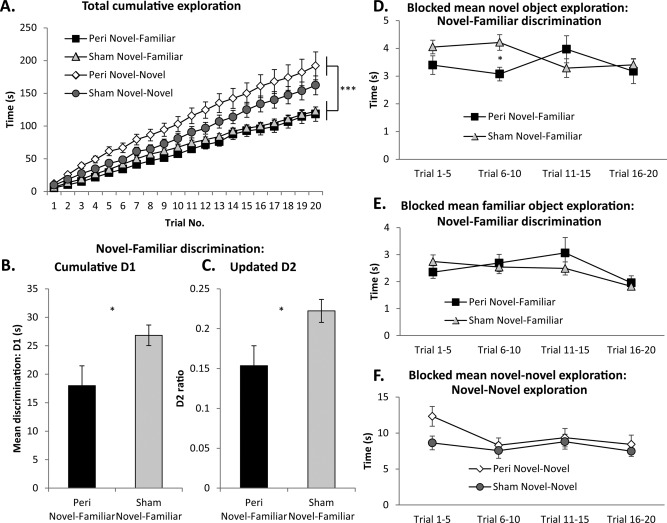
Behavioral measures for the two test conditions. A: Cumulative exploration times across the 20 test trials for all four groups. B, C: Discrimination performance of Cohort A, the Novel‐Familiar discrimination condition: cumulative D1 (B) and updated D2 ratio (C) following 20 trials. (These data are not available for the animals in the Novel‐Novel exploration condition as there was no discrimination to be performed). All discrimination scores are significantly above zero (one‐sample *t* tests, all *P* < 0.001). D: Mean exploration times of just novel objects of rats in Experiment 1 (Novel‐Familiar discrimination) blocked into four sets of five consecutive trials. E: Blocked mean exploration times of only familiar objects rats in Experiment 1 (Novel‐Familiar discrimination). F: Blocked mean exploration times of rats in Experiment 2 (Novel‐Novel exploration). *P < 0.05. Data presented are means ±SEM. Abbreviations: Peri, perirhinal cortex lesion.

Object exploration was video‐recorded, timed, and summed for both before and after an object was displaced to reach food. Exploration was defined as directing the nose at a distance <1 cm from the object with the vibrissae moving, and/or touching it with the nose or paws. Behavior not counted as exploration included sitting on the object, using the object to rear upward, and chewing the object. For the Novel–Familiar condition, two measures of discrimination were calculated (Fig. 3). Index D1 is the amount of time exploring the novel object, minus the time exploring the familiar object. The cumulative D1 is the sum of the D1 scores for all 20 trials. The second measure, D2 (Ennaceur and Delacour, 1988), compensates for individual differences in total exploration times. To calculate this index, the difference in time spent exploring the objects is divided by the total time spent in object exploration, i.e., D1 is divided by total object exploration. Consequently, the D2 ratio can range between −1 and +1. If the ratio is positive, the rat exhibits a preference for novel objects (recognition). The D2 score was recalculated after every trial using the cumulative amounts of exploration.

### Immunohistochemistry

On completion of testing and immediately prior to perfusion, rats were placed in a quiet dark room for 90 min. This interval matches the peak production of Fos (Bisler et al., 2002; Zangenehpour and Chaudhuri, 2002). The rats then received a lethal overdose of sodium pentobarbital (60 mg/kg, IP, Euthatal, Rhone Merieux) and were transcardially perfused with 0.1 M phosphate‐buffered saline (PBS) followed by 4% paraformaldehyde in 0.1 M PBS (PFA). The brains were removed and post‐fixed in PFA for 4 h, then incubated in 25% sucrose at room temperature overnight on a stirrer.

The brains were cut in the coronal plane into 40 µm sections using a freezing microtome. A 1 in 4 series of sections was collected in PBS, and then stained with cresyl violet. Immunohistochemistry was performed on another 1 in 4 series. This Fos staining was performed concurrently in pairs of rats, composed of perirhinal lesion and surgical control combinations. This procedure used a rabbit‐anti‐c‐Fos primary antibody (1 : 10,000; Cat #226 003; Synaptic Systems, Germany), biotinylated goat‐anti‐rabbit secondary antibody (1 : 200; Vector Laboratories), avidin‐biotinylated horseradish peroxidase complex (Elite kit, Vector Laboratories) all diluted in 0.2% Triton‐X in PBS and visualized by 3,3‐diaminobenzidine (DAB Substrate Kit, Vector Laboratories).

### Lesion Analysis

**Figure 4:**
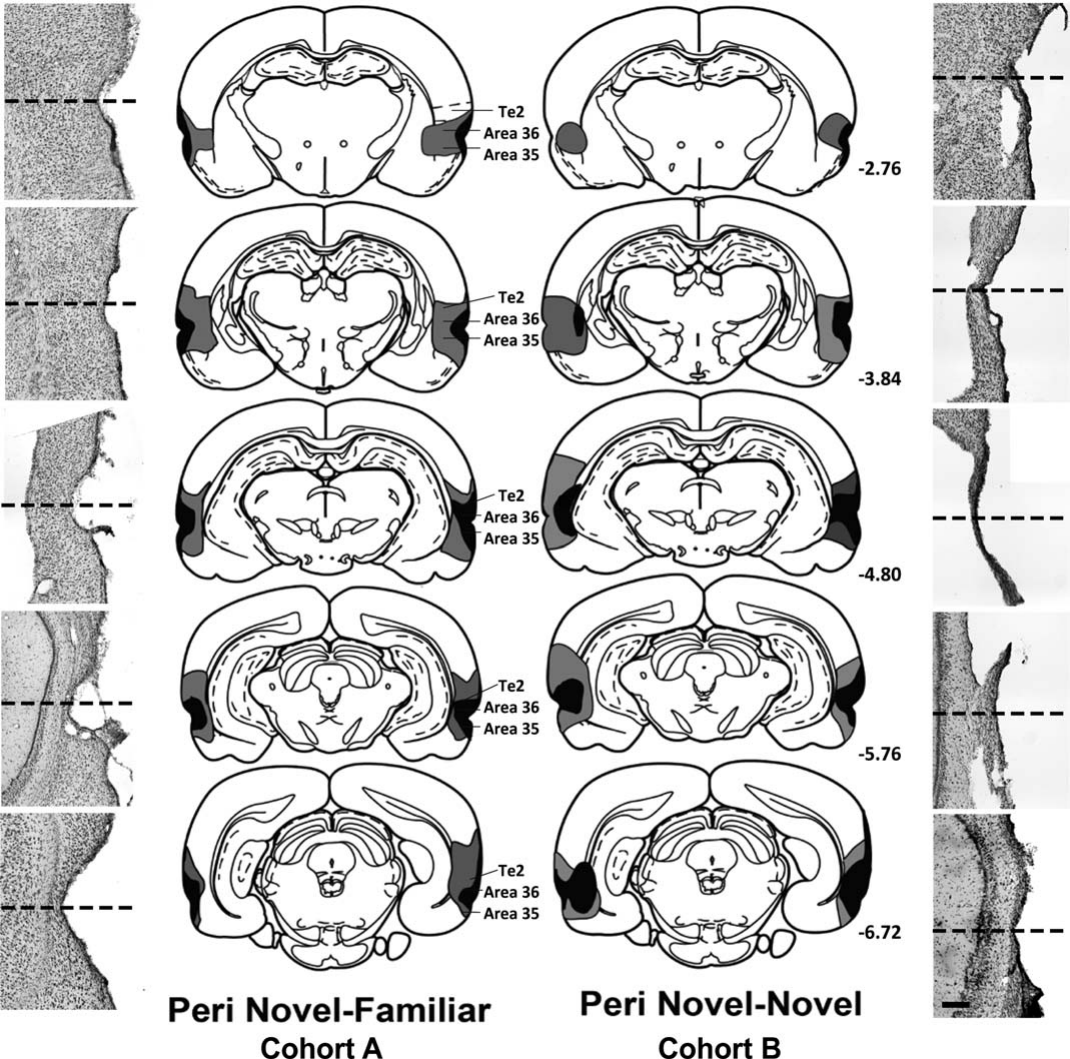
Diagrammatic reconstructions of the perirhinal cortex lesions (Peri) showing the individual cases with the largest (gray) and smallest (black) lesions for group Peri Novel‐Familiar from Cohort A (left; n = 12) and group Peri Novel‐Novel from Cohort B (right; n = 9). The numbers refer to the distance in millimeters from bregma (adapted from Paxinos and Watson, 2005). Outer panels are the corresponding cresyl violet stained sections taken from the rat with the median sized lesion in each cohort. The dashed line represent the location of the rhinal sulcus. Scale bar 200 μm.

The perirhinal cortex lesions were reconstructed from the Nissl stained sections. Each lesion was plotted onto five coronal plates, each ∼1 mm apart (Fig. 4). The total extent of perirhinal cortex damage was calculated from these reconstructions, using the perirhinal borders of Burwell (2001). A hemisphere was excluded if there was evidence of hippocampal cell disruption in two adjacent sections (120‐µm apart). Cases with damage to both hippocampi were completely removed from the study. These exclusions were made prior to cell counting.

### Fos‐Positive Cell Counts

Digital images were captured from all regions of interest (ROI) in the selected hemispheres (see above) using a Leica DMRB microscope and an Olympus DP73 Camera. Six consecutive sections (each 120µm apart) were taken from each ROI. Immunopositive cells were counted using CellSens Dimensions software (Olympus, UK) to avoid experimenter bias. Fos‐positive cells were defined as having immunopositive nuclei (diameter of 4 − 20 µm, sphericity of 0.1 − 1.0) stained above a grayscale threshold set at 50 − 60 units below the peak gray value measured by a pixel intensity histogram. The experimenter remained blind to the group conditions.

While stereological methods are essential to derive accurate, absolute cell counts (Coggeshall and Lekan, 1996), this study sought to compare relative Fos‐positive counts between animal groups and areas. For this purpose, automated cell counting is appropriate when certain conditions are met (Coggeshall and Lekan, 1996; Mura et al., 2004). These conditions include no systematic changes in the volume or packing of the neurons across the animal groups, along with random tissue sampling. To test the former, the number of cells with a diameter of 4 − 20 µm per unit area (µm^2^) was calculated in the caudal lateral entorhinal cortex using the Nissl stained sections. Lateral entorhinal cortex was selected as it is adjacent to perirhinal cortex and receives dense inputs from that area, making it most likely to be affected by the loss of perirhinal tissue.

### Regions of Interest

**Figure 5:**
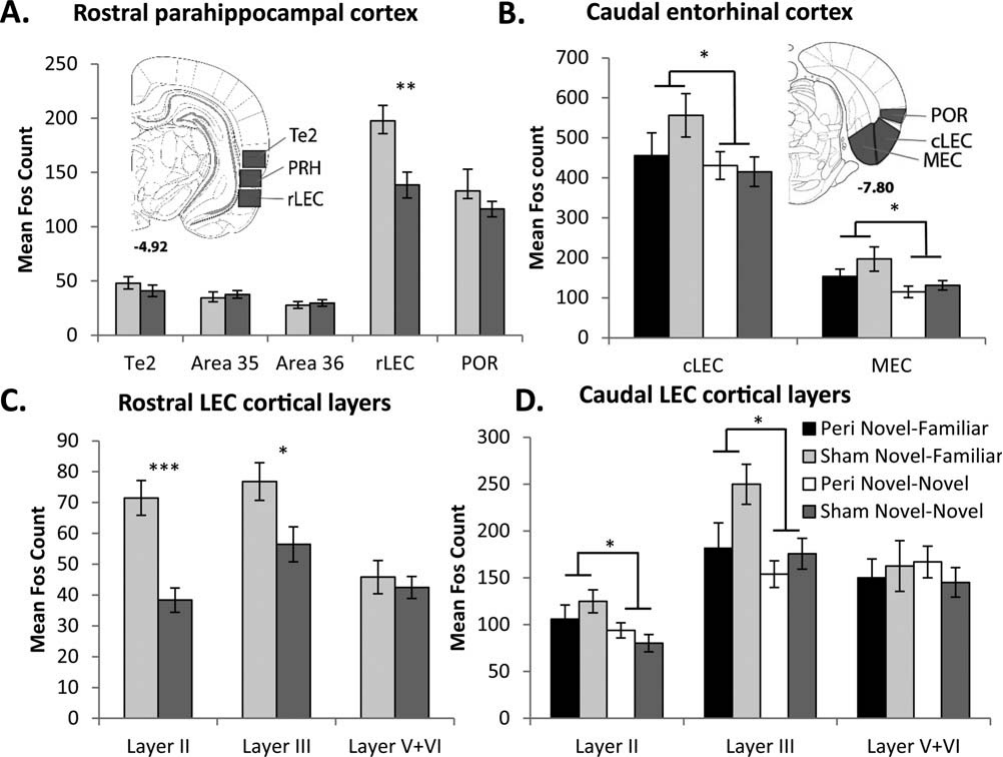
Graphs depict mean Fos counts for four regional groupings of the entorhinal cortex and adjacent cortical regions. Inset: illustrations of regions of interest; the numbers refer to the distance in millimeters from bregma; adapted from the atlas of Paxinos and Watson (2005). Abbreviations: cLEC, caudal lateral entorhinal cortex; MEC, medial entorhinal cortex; POR, postrhinal; PRH, perirhinal cortex; rLEC, rostral lateral entorhinal cortex; V + VI, cortical Layers V and VI combined. *P < 0.05; **P < 0.01; ***P < 0.001. Data are presented as means ±SEM.

**Figure 6:**
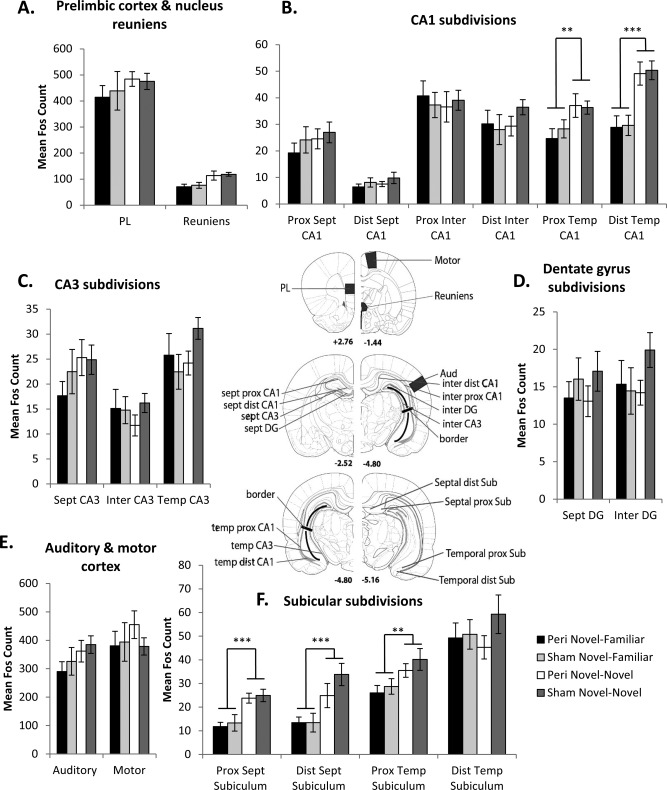
Graphs that illustrate mean Fos counts in the prelimbic cortex and nucleus reuniens of the thalamus (A), control regions (E) and the hippocampal formation (B, C, D, F), with regions of interest indicated in the central panel. The numbers in the central panel refer to the distance in millimeters from bregma. Panel −4.80 is repeated to help depict all analyzed regions. [Adapted from the atlas of Paxinos and Watson (2005).] Abbreviations: Aud, primary auditory cortex; DG, dentate gyrus; Dist, distal; Inter, intermediate; Motor, primary motor cortex; PL, prelimbic cortex; Prox, proximal; Reuniens, nucleus reuniens of the thalamus; Sept, septal; Sub, subiculum; Temp, temporal. Data presented are means ±SEM.

Figures 5 and 6 depict the multiple regions of interest (ROIs). The numbers give the anterior – posterior (AP) coordinates of the ROIs, relative to bregma (see Paxinos and Watson, 2005).

#### Parahippocampal cortex—Sham animals only

In the sham surgical groups only, Fos‐positive cell counts were made in the caudal half of areas 35 (ventral) and 36 (dorsal) of perirhinal cortex (Burwell, 2001), as well as the adjacent area Te2 and rostral lateral entorhinal cortex (rLEC) (AP −4.80 to −5.52) (Fig. 1). [The rLEC is also described as the dorsal intermediate entorhinal (DIE) field (Insausti et al., 1997)]. Separate counts were made in Layers II, III, and V + VI (combined) of the rLEC (Fig. 1). These laminar subdivisions reflect how LEC cortical Layer II preferentially projects to the dentate gyrus and CA3, while LEC layer III projects to CA1. The reciprocal projections from the hippocampus predominantly terminate in the deeper layers (V + VI) of entorhinal cortex (Steward and Scoville, 1976; Amaral, 1993; Tamamaki and Nojyo, 1995). The postrhinal cortex (POR) was analyzed from sections corresponding to AP −7.08 to −8.04. Two parahippocampal areas (rLEC and POR) were not analyzed in the lesion groups due to the presence of occasional extra perirhinal damage (see below).

#### Caudal entorhinal cortex—All animals

Cell counts were taken from the medial entorhinal cortex (MEC) as well as a more caudal region of LEC (cLEC) from AP −7.08 to −8.04 (Fig. 5). The area boundaries correspond to those of Burwell and Amaral (1998). Separate counts were again made in cortical Layers II, III, and V + VI (combined) of the cLEC (see Fig. 1).

#### Hippocampal formation

Hippocampal subfields (dentate gyrus, CA1, and CA3) were divided into their septal (dorsal), intermediate, and temporal (ventral) divisions (Bast, 2007; Strange et al., 2014; see Fig. 6). The CA1 subfield was further subdivided into its proximal and distal (relative to DG) halves. The dorsal and ventral blades of the dentate gyrus were initially analyzed separately but counts were collapsed when this separation did not alter the pattern of results. The septal hippocampus counts (dentate gyrus, CA3 and CA1) were obtained from AP −2.52 to −3.24, while those for the intermediate hippocampus (dentate gyrus, CA1, CA3) came from sections near AP −4.80 to −5.52. The border between the intermediate and temporal hippocampus corresponds to −5.0 dorsoventral from bregma (Paxinos and Watson, 2005). Within the temporal (ventral) hippocampus, counts were made in the CA1 and CA3 fields from approximately AP −4.80 to −5.52. Additional cell counts were taken in both the septal and temporal subiculum (from around AP −5.16). As with CA1, the subicular divisions were also further subdivided into proximal and distal halves.

#### Prelimbic cortex, auditory cortex, motor cortex, and thalamus

Fos‐positive cell counts were made within the prelimbic cortex (PL) (from AP +4.20 to +2.76) and nucleus reuniens of the thalamus (from AP −1.44 to −2.52). For control purposes, counts of Fos‐positive cells were made in the primary auditory cortex (from AP −4.80 to −5.52) and the primary motor cortex (from AP −0.60 to −1.60), regions that should not differentiate the two behavioral tasks.

### Statistical Analyses

#### Behavior

For the Novel–Familiar discrimination (Cohort A), total exploration times (two‐sample *t*‐test, two‐tailed), cumulative D1 and updated D2 scores (two‐sample *t*‐tests, one‐tailed) were compared between the perirhinal cortex and control groups. Additional one‐sample *t*‐tests (one‐tailed) on the final cumulative D1 and updated D2 scores assessed whether discrimination performance was above chance (zero). For both behavioral conditions, the exploration times for each session were further analyzed by dividing them into four blocks of five consecutive trials. An ANOVA then compared the perirhinal lesion and control groups, with the additional within‐subject factor of block.

#### IEG analyses

Where appropriate, cell counts were grouped by area or subfield. These groupings reduce the number of comparisons and, thereby, restrict Type 1 errors. ANOVAs compared different components of a region (e.g., septal or temporal, proximal or distal), as well as surgical condition (perirhinal lesion or sham). When an interaction was significant, the simple effects were examined. Finally, inter‐regional Pearson product‐moment correlation coefficients were calculated for the Fos‐positive cell counts in all sites, within each of the four conditions. These correlations provide the first step for structural equation modelling. Inter‐regional Fos correlations were compared between groups using Fisher's r‐to‐z transformation (Zar, 2010).

### Structural Equation Modeling (SEM)

Structural equation modelling evaluates the viability (fit) of network dynamics (McIntosh and Gonzalez‐Lima, 1991; Friston et al., 1993; Jenkins et al., 2003; Poirier et al., 2008). As recommended, several goodness of fit measures are reported (Fan and Wang, 1998; Hu and Bentler, 1998; Tabachnik and Fidell, 2001). The first indication of a model of good fit is a non‐significant chi‐square (*χ^2^*), which gives a binary fit/no‐fit decision for the model. The comparative fit index (CFI) is based on the comparison of the proposed model to an independent model where there is no relationship between any anatomical regions. A high index value means that the tested model is opposite to the independent model, indicating good fit (acceptable CFI ≥ 0.9). The third measure is the root mean square error of approximation (RMSEA), which provides an index of absolute fit as it estimates the square root of the mean lack of fit per degree of freedom. Thus, it can account for parsimony in the model. An RMSEA <0.1 is considered acceptable.

Both the CFI and RMSEA are appropriate indices for studies with relatively small sample sizes (Fan and Wang, 1998; Hu and Bentler, 1998). Additionally, to ensure these model fit statistics remained robust with small sample size, the ratio of regions specified in each model to the number of cases was held at a maximum of two for every model tested (Wothke, 1993). The SEM specialized software SPSS AMOS 20.0 was used (IBM Corp, Armonk, NY). A direction of effect could not be inferred between some anatomical regions as the fit of the models did not change when the path direction was reversed. This condition is indicated in the figures by a double‐headed arrow. Additionally, the squared multiple correlation (*R* ^2^ or coefficient of determination) is presented for each endogenous brain region in the models. This value is a measure of the proportion of its variance that can be explained by its inputs within the model (Arbuckle, 2011).

The data from different groups were then compared by a stacking procedure that tests for group differences in the path coefficients within the same overall model (Protzner and McIntosh, 2006; Schumacker and Lomax, 2010). Path coefficients are regression weights that indicate the strength of the relationship between regions. For stacking, the path coefficients of all paths in the model are constrained to be equal for all groups, then each path is independently unconstrained and the fit compared to that of the model in which all paths are constrained (structural weights model). If the model fit when the path is unconstrained is significantly improved, as determined by a *χ* ^2^‐difference test, this indicates that the strength of that path differs among the groups.

## RESULTS

### Histology: Perirhinal Lesion Analyses

#### Cohort A—Novel‐Familiar discrimination

Fos‐positive cell counts were taken from one hemisphere per animal and were restricted to those hemispheres with no hippocampal damage or only very limited hippocampal cell loss confined to just one coronal section. According to these criteria, five of the seventeen rats with perirhinal lesions were rejected. Thus, final group sizes were Perirhinal Novel‐Familiar, *n* = 12 and Sham Novel‐Familiar, *n* = 12. Perirhinal tissue damage across both hemispheres in these twelve rats ranged from 53.7% to 97.6% (mean 73.7%). The lesions typically involved almost the full anterior‐posterior extent of areas 35 and 36 (Fig. 4). A frequent feature was the encroachment of the lesion into the most dorsal parts of the piriform cortex and the rostral regions of LEC (Fig. 4, left panel), i.e., those cortices adjacent to area 35. A total of five left hemispheres and seven right hemispheres were analyzed for c‐*fos* in the Perirhinal group. Corresponding hemispheres were analyzed in the Sham control group.

#### Cohort B—Novel‐Novel exploration

Nine of the eighteen rats with perirhinal lesions were rejected according to the histological exclusion criteria. One surgical control rat was eliminated due to the presence of idiopathic damage in the left frontotemporal cortex. Final group sizes were, therefore, Perirhinal Novel‐Novel, *n* = 9 and Sham Novel‐Novel, *n* = 12. Perirhinal damage across both hemispheres ranged from 63.9% to 98.3% (mean 82.8%). The appearance of the lesions in Cohort B matched those in Cohort A (Fig. 4, right panel), with no group difference in overall perirhinal tissue loss (*t*_19_ = 1.68, *P* = 0.11). A total of two left hemispheres and seven right hemispheres were analyzed for Fos in the Perirhinal lesion group. Both the behavioral and IEG data presented below were obtained from the same (non‐rejected) animals.

### Behavior: Perirhinal Cortex Lesions and Recognition Memory (Cohort A: Novel–Familiar Discrimination)

Recognition memory was tested using the bow‐tie maze procedure (Fig. 2). Briefly, this utilizes a running recognition protocol in which the novel object in one trial becomes the familiar object in the subsequent trial (Albasser et al., 2011a). Rats received 20 trials. Removal of the perirhinal cortex significantly reduced the rats' preference for novel over familiar objects but did not entirely eliminate this discrimination at the very short retention intervals used in the present study (<1 min). Consequently, both recognition indices D1 and D2 were significantly lower in the Perirhinal group than the Sham controls (cumulative D1, *t*_22_ = 2.26, *P* = 0.017; updated D2, *t*_22_= 2.38, *P* = 0.014; Figs. 3B,C), although both groups still discriminated above chance levels (D1, Perirhinal, *t*_11_ = 5.20, *P* < 0.001; Sham, *t*_11_ = 14.8, *P* < 0.0001; D2, Perirhinal, *t*_11_ = 6.11, *P* < 0.0001; Sham, *t*_11_ = 15.4, *P* < 0.0001; Figs. 3B,C). Despite the discrimination deficit, there was no lesion difference in the total time spent exploring objects (*t*_22_ = 0.34, *P* = 0.73; Fig. 3A).

### Behavior: Perirhinal Cortex Lesions and the Exploration of Novel Objects (Cohort B: Novel–Novel Exploration)

Cohort B explored pairs of dissimilar, novel objects in the bow‐tie maze (Fig. 2), consequently no recognition indices could be calculated. The focus was, therefore, on the total exploration times from each trial. Perirhinal lesions did not affect the times spent exploring novel objects when compared with their Sham controls (*F*_1,19_ = 1.50, *P* = 0.24; Fig. 3F). When the exploration data were separated into four blocks of five trials to test if exploration levels changed across the test session (Fig. 3F) there was a significant effect of block, as the first block attracted the most attention (*F*_3,57_ = 5.68, *P* = 0.002). However, this enhancement was not differentially affected by the surgical status of the rats as there was no lesion by block interaction (*F*_3,57_ = 2.16, *P* = 0.10). Likewise, when the total [novel objects (Fig. 3D) plus familiar objects (Fig. 3E)] exploration times for Cohort A were grouped into four consecutive blocks, again, there was a significant effect of block (*F*_3,66_ = 3.73, *P* = 0.015) but no overall effect of lesion (*F* < 1). There was, however, an interaction between these terms (*F*_3,66_ = 3.31, *P* = 0.025). This interaction did not reflect differences between the lesions and their surgical controls during any individual block (all *F*_1,22_ < 2.5, *P* > 0.13), rather that the Sham Novel‐Familiar group reduced their level of exploration as blocks progressed (*F*_3,20_ = 3.66, *P* = 0.030) while the Perirhinal Novel‐Familiar group did not (*F*_3,20_ = 2.84, *P* = 0.064).

The two behavioral conditions were then further compared. Consistent with the expected greater exploration of novel objects, the Novel‐Novel condition (Cohort B) promoted more total exploration time than the condition with one novel and one familiar object (Novel‐Familiar, Cohort A) (*F*_1,41_ = 18.4, *P* < 0.001; Fig. 3A). This increased exploration was not affected by perirhinal lesions as there was no overall group difference (*F* < 1) and no interaction between behavioral condition and lesion (*F*_1,41_ = 1.67, *P* = 0.20).

### Histology: Cell Density in the Caudal Lateral Entorhinal Cortex (cLEC)

Before comparing Fos‐positive cell counts, we examined whether the perirhinal lesions affected cell density (cell number/µm^2^) in a closely related site (cLEC). Any such changes would compromise the Fos counts. The area was selected as it is immediately adjacent to the lesions and strongly interconnected with perirhinal cortex. (Rostral LEC (rLEC) was not examined as the perirhinal lesions sometimes encroached directly into this area.) Comparisons, based on the Nissl stained sections, revealed that the perirhinal lesions did not alter cLEC cell density (*F* < 1), nor did the behavioral condition (*F* < 1), with no interaction between these terms (*F* < 1). [The respective cell densities in cLEC were as follows: Perirhinal Novel‐Familiar 1.80 x 10^−3^ ± 6.58 x 10^−5/^µm^2^; Sham Novel‐Familiar 1.76 x 10^−3^ ± 4.39 x 10^−5/^µm^2^; Perirhinal Novel‐Novel 1.77 x 10^−3^ ± 8.16 x 10^−5/^µm^2^; Sham Novel‐Novel 1.75 x 10^−3^ ± 2.88 x 10^−5^/µm^2^.] This analysis was based on cells of diameter 4 − 20 µm as this range corresponds to that used for the Fos counts. In addition, cLEC cells in the range of 1 − 3.99 µm and 20.01 − 50 µm were also counted and, again, there were no lesion or condition differences in cell density (all *F* < 1). These results indicate that the lesions did not cause systematic changes in cell packing in a region immediately adjacent to the lesion site that would normally receive numerous perirhinal inputs.

### c‐*Fos* Imaging: Behavioral Control Comparisons

Prior to contrasting Cohorts A and B, it was necessary to determine if the two behavioral tasks were matched for sensorimotor demands. Fos‐positive cell counts were, therefore, made in two pre‐selected areas (the primary auditory and primary motor cortices). No overall difference in Fos‐positive cells was found for the two behavioral conditions (*F*_1,41_ = 1.26, *P* = 0.27; Fig. 6E) or for lesion status (*F* < 1), with no significant interaction (*F* < 1). Further, the two cortical Fos‐counts did not significantly interact with the behavioral condition (*F* < 1) or with lesion status (*F*_1,41_ = 2.60, *P* = 0.12). Finally, the three‐way interaction was not significant (*F*_1,41_ = 1.04, *P* = 0.31). These findings help suggest that the behavioral conditions were appropriately matched.

### c‐*Fos* Imaging: Effects of Behavioral and Surgical Conditions

The study involved 26 ROIs and four animal groups. Each hippocampal subfield was analyzed separately, incorporating its separate cell counts for proximal‐distal and septal‐temporal divisions. Other than hippocampal subfields, there is no reason to suppose that two different brain regions may have comparable activity levels for a particular task. Thus, to simplify the analyses, Fos count comparisons between regions (i.e., the main effect of region in each ANOVA) are not presented. Any such area effects could reflect different basal activity levels. Consequently, regional differences are only presented if an area's Fos‐related activity significantly interacts with the behavioral task or with surgical status, assuming this analysis is not confounded by scaling effects. Regional groupings, which helped to compensate for Type 1 errors, were based on shared anatomical or network features. An example concerns how prelimbic cortex is densely and reciprocally connected with nucleus reuniens of the thalamus (Vertes et al, 2007), creating indirect links with temporal CA1. A brief summary of the main findings begins each results section.

### Parahippocampal Cortices and Te2 – Behavioral Effects (Sham Groups Only)

The main difference in the parahippocampal cortices (perirhinal, postrhinal, and rostral LEC) and area Te2 of the two control groups was higher Fos counts in rostral LEC (rLEC) in the Novel‐Familiar discrimination condition over that associated with Novel‐Novel exploration. Further analyses localized this Fos increase to lateral entorhinal cortical Layers II and III.

While the overall number of Fos‐positive neurons in the parahippocampal cortices and area Te2 of the surgical control rats did not distinguish the Novel–Novel and Novel–Familiar conditions (*F*_1,22_ = 2.31, *P* = 0.14; Fig. 5A), a significant region by behavioral condition interaction was found (*F*_4,88_ = 6.55, *P* < 0.001). This interaction reflected higher Fos expression in the rostral part of the LEC by the Sham Novel‐Familiar discrimination group than group Sham Novel‐Novel (*F*_1,22_ = 10.1, *P* = 0.004).

The numbers of Fos‐positive cells in rLEC were separated into cortical Layers II, III, and V + VI (combined), reflecting their different hippocampal connections (see Figs. 1 and 5). The first question was whether the Novel‐Familiar object discrimination caused a general or lamina specific increase in rLEC activity (Fig. 5C). This analysis revealed higher Fos counts in rLEC for the discrimination task (*F*_1,22_ = 9.51, *P* = 0.005) as well as a significant behavioral condition by layer interaction (*F*_2,44_ = 9.87, *P* = <0.001). This interaction reflected higher Fos expression associated with the Novel‐Familiar discrimination condition in hippocampal input cortical Layer II (*F*_1,22_ = 23.3, *P* < 0.001) and Layer III (*F*_1,22_ = 5.96, *P* = 0.023), but not the deeper cortical layers (*F* < 1).

### Caudal Entorhinal Cortex—Surgical and Behavioral Effects (All Four Groups)

As was observed in the more rostral entorhinal area, the caudal entorhinal region of the Novel–Familiar discrimination condition had higher Fos‐positive cell counts than the Novel–Novel exploration condition. Again, this behavioral condition effect was restricted to the superficial entorhinal cortical layers. Additionally, perirhinal cortex lesions attenuated Fos‐expression specifically in cortical Layer III of caudal LEC (cLEC).

Formal comparisons revealed no overall effect of perirhinal lesions on Fos‐positive cell counts in cLEC or medial entorhinal cortex (MEC) (*F*_1,41_ = 1.22, *P* = 0.28), nor did the lesions differentially affect the two behavioral conditions (*F*_1,41_ = 1.17, *P* = 0.27). There was, however, a significant main effect of behavior (*F*_1,41_ = 4.22, *P* = 0.046) as higher Fos counts were seen in both MEC and cLEC following the Novel‐Familiar discrimination condition, regardless of surgery (Fig. 5B). There were no two‐way interactions with surgery or area (both *F* < 1), nor was the three‐way interaction significant (*F*_1,41_ = 1.46, *P* = 0.23).

Specific entorhinal laminar activity was then compared to see if the lamina difference in rLEC for the two behavioral conditions (see above, Fig. 5C) generalized to the cLEC. There was no overall effect of the behavioral condition on the cLEC cortical layers (*F*_1,41_= 2.87, *P* = 0.098), no effect of perirhinal surgery (*F* < 1), nor a behavior by lesion interaction (*F*_1,41_ = 1.47, *p* = 0.23; Fig. 5D). The cortical layers were, however, differently modified by the behavioral condition (*F*_2,82_ = 4.04, *P* = 0.021) as rats in the Novel‐Familiar discrimination condition had significantly higher Fos counts in hippocampal input cortical Layer II (*F*_1,41_ = 5.43, *P* = 0.025) and Layer III (*F*_1,41_ = 5.65, *P*= 0.022), but not the deeper entorhinal layers (*F* < 1; Fig. 5D). Again, these interactions suggest that active discrimination leads to greater activity in hippocampal input layers compared to novel object exploration. Finally, the layer by lesion interaction was significant (*F*_2,82_= 4.52, *P* = 0.014). Simple effects demonstrated that perirhinal lesions reduced the number of Fos‐positive cells in Layer III (*F*_1,41_ = 4.42, *P* = 0.042) but not Layer II or V + VI (*F* < 1 for both comparisons), regardless of the behavioral condition.

### Hippocampal Formation

#### CA1

Fos imaging revealed that the temporal CA1 is sensitive to the quantity of novel stimuli. Increased Fos counts were observed in both the proximal and distal areas of temporal CA1 associated with the Novel‐Novel exploration condition (Fig. 6B), an effect not modified by the lesions status of the rats.

Statistical comparisons revealed that perirhinal cortex lesions did not affect overall Fos levels across CA1 (*F* < 1; Fig. 6B), nor did the behavioral task (*F*_1,41_= 3.74, *P* = 0.06), and there was no interaction between these terms (*F* < 1). There was, however, a significant interaction between septotemporal level and the behavioral task (*F*_2,82_ = 14.0, *P* < 0.001) as Novel‐Novel object exploration resulted in relatively increased Fos expression over Novel‐Familiar discrimination in temporal CA1 (*F*_1,41_ = 17.6, *P* < 0.001), but not in the septal or intermediate levels (*F*_1,41_ = 1.02, *P* = 0.32; *F* < 1 respectively; see Fig. 6B).

The interaction between the proximal‐distal and septotemporal dimensions was not modified by perirhinal cortex lesions (*F*_2,82_ = 1.12, *P* = 0.34), though it was altered by the behavioral condition (*F*_2,82_ = 5.28, *P* = 0.007). The two behavioral tasks did not cause differences along the proximal‐distal axis of CA1 at septal and intermediate levels (*F* < 1), whereas in temporal CA1 both the proximal and distal regions displayed higher Fos expression when the rats explored Novel‐Novel objects as compared to the Novel‐Familiar discrimination condition (proximal: *F*_1,41_ = 8.52, *P* = 0.006 and distal: *F*_1,41_ = 25.8, *P* < 0.001). The four‐way interaction was not significant (*F* < 1).

#### CA3

Fos counts in CA3 were not affected by the behavioral task (*F*_1,41_ = 1.01, *P* = 0.32) or by lesion status (*F* < 1). Similarly, there no interaction between these two factors (*F* < 1; Fig. 6C). Differences were seen across the septotemporal levels of CA3 (*F*_2,82_ = 21.3, *P* < 0.001), with Fos counts lowest in intermediate CA3, but these effects were not altered by behavior (*F*_2,82_ = 1.51, *P* = 0.23), perirhinal cortex lesions (*F* < 1), or their combination (*F*_2,82_ = 2.40, *P* = 0.097).

#### Dentate gyrus

Fos counts in the dentate gyrus appeared insensitive to the various manipulations. Consequently, there was no effect of behavioral condition (*F* < 1) or of perirhinal lesions (*F*_1,41_ = 1.41, *P* = 0.24), with no interaction (*F* < 1; Fig. 6D). Likewise, there were no Fos count differences between the septal and intermediate dentate gyrus (*F* < 1), nor any other significant main effects or interactions (all *P* > 0.25). Separating the Fos counts between the upper and lower blades of the dentate gyrus did not alter the pattern of results.

#### Subiculum

The main observation in the Sham control groups was an increase in Fos counts associated with the Novel‐Novel condition compared to the Novel‐Familiar discrimination condition in all subicular sub‐regions examined except the distal temporal subiculum. In the perirhinal lesion groups, Novel‐Novel exploration increased Fos‐expression in the proximal septal and proximal temporal areas of the subiculum, but not the adjacent distal areas. Consequently, the perirhinal lesions appeared to affect the distal subiculum in a task specific manner, that is, in lesion animals Fos‐related activity in that subregion did not increase when rats explored only novel objects.

As with other hippocampal areas, there was no overall effect of perirhinal cortex lesions on Fos expression across the subiculum (*F*_1,41_ = 1.63, *P* = 0.21; Fig. 6F). There was, however, a main behavioral effect as Novel‐Novel object exploration produced higher subicular Fos counts than Novel‐Familiar discriminations (*F*_1,41_= 8.98, *P* = 0.005; Fig. 6F). This behavioral effect was not differentially affected by perirhinal lesions (*F* < 1).

The interaction between the septotemporal level and the proximal and distal sub‐regions was modified by the behavioral task (*F*_1,41_ = 5.88, *P* = 0.02) but not by the perirhinal surgeries (*F* < 1). The Novel‐Novel condition had higher Fos counts than Novel‐Familiar discrimination in proximal septal subiculum (*F*_1,41_ = 19.4, *P* < 0.001), distal septal subiculum (*F*_1,41_ = 15.2, *P* < 0.001) and proximal temporal subiculum (*F*_1,41_ = 8.03, *P* = 0.007), but not distal temporal subiculum (*F* < 1; Fig. 6F). Thus, the distal temporal subiculum did not increase Fos expression when exposed to novel‐novel objects regardless of lesion status.

Additionally, there was a significant three‐way interaction between the proximal and distal sub‐regions, the behavioral condition, and lesion status (*F*_1,41_= 4.62, *P* = 0.037). In the surgical control animals, the Novel‐Novel condition was associated with higher Fos counts than the Novel‐Familiar discrimination condition in both the proximal (*F*_1,41_ = 10.7, *P* = 0.002) and distal (*F*_1,41_ = 6.03, *P* = 0.018) regions of the subiculum. While in the perirhinal lesion groups, higher Fos counts were associated with the proximal (*F*_1,41_ = 8.01, *P* = 0.007) but not the distal region in the Novel‐Novel condition (*F* < 1; Fig. 6F). Finally, the four‐way interaction was not significant (*F* < 1).

### Prelimbic Cortex and Nucleus Reuniens of the Thalamus

Fos‐positive cell counts in the prelimbic cortex and nucleus reuniens of the thalamus were not affected by behavioral task (*F*_1,41_ = 2.74, *P* = 0.11; Fig. 6A) or lesions status (*F* < 1), nor was the interaction significant (*F* < 1). Similarly, neither the behavioral task nor the perirhinal cortex lesions differentially affected these two regions (*F* < 1 for all interaction terms).

### Structural Equation Modeling

When Fos‐positive group means for multiple brain regions remain comparable, the underlying inter‐regional relationships may be markedly different (e.g., Poirier et al., 2008). Thus, structural equation modelling techniques were employed to assess whether network dynamics were altered by surgical or behavioral conditions.

All network models were based on known patterns of medial temporal lobe connectivity. A further constraint was to base the analyses on already published models with good fit. In this way, the reliability of previous models was tested. These network analyses were based on inter‐area correlations of Fos counts. Inspection of these correlation matrices (correlation coefficients between each region with all of the other regions) immediately revealed that the Novel‐Familiar discrimination task was associated with a much higher number of significant (uncorrected, *P* ≤ 0.05) inter‐area Fos count correlations than the Novel‐Novel condition. For each of the two Sham groups there were a total of 465 possible inter‐area correlations. For group Sham Novel‐Familiar, ∼69% (that is 320/465) of the possible inter‐region correlations reached significance (*p* ≤ 0.05), while for the Sham Novel‐Novel group only 13% of the possible correlations (63/465) were significant.

**Figure 7:**
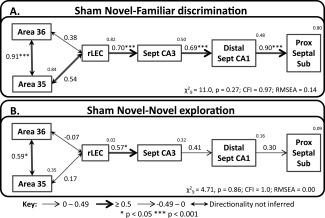
Structural equation models for novel object processing. The figure shows the networks with best fit for the Sham Novel‐Familiar (A) and Sham Novel‐Novel (B) groups. The fit is noted under each model (CFI, comparative fit index; RMSEA, root mean square error of approximation). The strength of the causal influence of each path is denoted both by the thickness of the arrow and by the path coefficient next to that path. The number above each region is the proportion of its variance that can be explained by its inputs. Sites depicted: area 35 and area 36 of the caudal perirhinal cortex, rostral lateral entorhinal cortex (rLEC), septal CA3, distal septal CA1, and septal proximal subiculum. *P < 0.05; ***P < 0.001.

**Figure 8:**
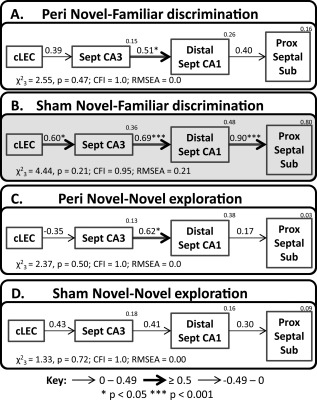
Depictions of the caudal parahippocampal ‐ hippocampal interactions derived by structural equation modelling for groups; Peri Novel‐Familiar (A), Sham Novel‐Familiar (B), Peri Novel‐Novel (C) and Sham Novel‐Novel (D). The fit is noted under each model (CFI, comparative fit index; RMSEA, root mean square error of approximation) and models with unacceptable fit are represented with a pale gray background. The strength of the causal influence of each path is denoted both by the thickness of the arrow and by the path coefficient next to that path. The number above each region is the proportion of its variance that can be explained by its inputs. Sites depicted: caudal lateral entorhinal cortex (cLEC), septal CA3, distal septal CA1, and septal proximal subiculum. *P < 0.05; ***P < 0.001.

**Figure 9:**
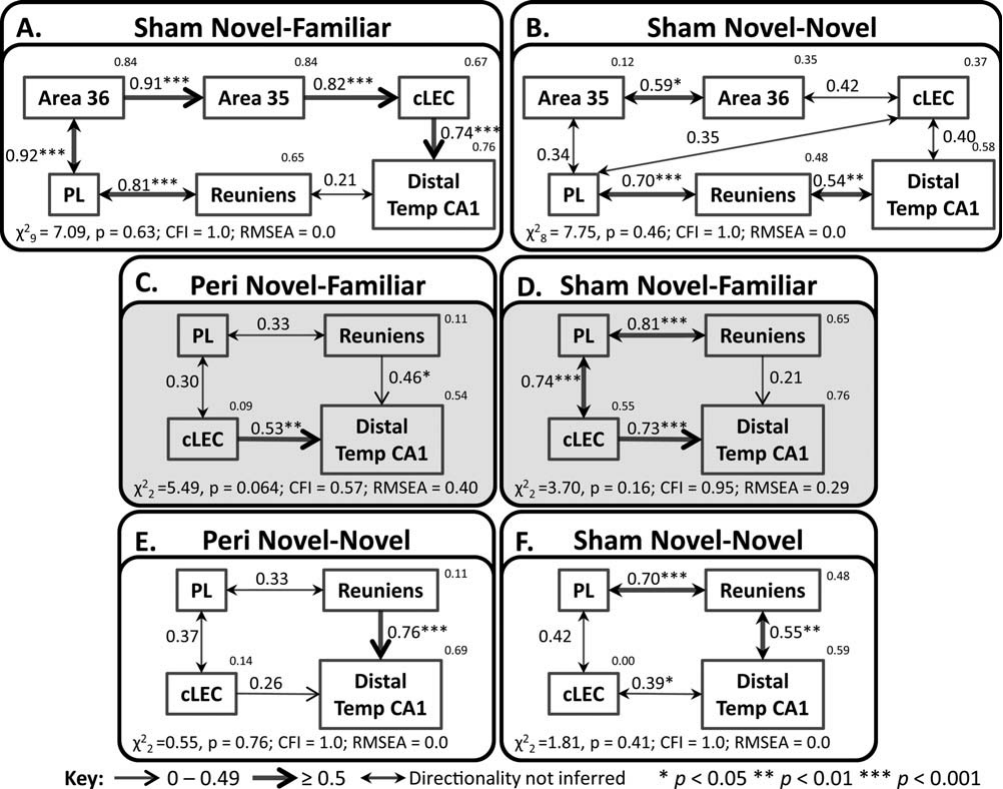
The upper panels depict the optimal interactions between prelimbic cortex, the rhinal cortex and temporal hippocampus derived by structural equation modeling for the surgical control groups; Sham Novel‐Familiar (A), and Sham Novel‐Novel (B). The lower panels illustrate similar interactions excluding perirhinal cortex for groups; Peri Novel‐Familiar (C), Sham Novel‐Familiar (D), Peri Novel‐Novel (E) and Sham Novel‐Novel (F). The fit is noted under each model (CFI, comparative fit index; RMSEA, root mean square error of approximation) and models with unacceptable fit are represented with a pale gray background. The strength of the causal influence of each path is denoted both by the thickness of the arrow and by the path coefficient next to that path. The number above each region is the proportion of its variance that can be explained by its inputs. Sites depicted: Areas 35 and 36 of the perirhinal cortex, caudal lateral entorhinal cortex (cLEC), distal temporal CA1, prelimbic cortex (PL), and nucleus reuniens of the thalamus. *P < 0.05; **P < 0.01; ***P < 0.001.

A model is referred to in the following sections as having good fit to the observed data when certain criteria are met. These criteria include a nonsignificant chi‐square (*χ* ^2^) with a ratio of *χ* ^2^ to the associated degrees of freedom of <2. Additionally, a CFI measure of > 0.9 and/or an RMSEA of <0.1 is required (see Methods for further details). Where possible, these measures are presented in Figures 7, 8, 9 and so the goodness of fit indices are given in the text only for those models not depicted in the figures.

### A Network for Novel Objects—Sham Animals Only

The initial SEM analyses used the best‐fitting model from previous, comparable tests of recognition memory (Albasser et al., 2010; Kinnavane et al., 2014). This model involves perirhinal cortex, rLEC, and the septal hippocampus. For these reasons, the first analyses considered just the Sham Novel‐Familiar object group, i.e., the recognition memory condition. An added refinement was to incorporate the preferential projections of the LEC to distal CA1 and proximal subiculum, i.e., further divide the data along the proximal‐distal axis (Amaral, 1993; Witter, 1993).

The perirhinal cortex was divided into its composite areas (areas 35 and 36), creating a model with good indices of fit for the Sham Novel‐Familiar group (Fig. 7A). The resulting model involved parallel projections between area 36 of the perirhinal cortex and rLEC; one direct, the other via area 35 (Fig. 7A). Thereafter, the route followed previously defined, but more anatomically refined, routes, i.e., rLEC to septal CA3, to septal CA1 (distal) and, thence, to the septal subiculum (proximal; Fig. 7A). Altering the model so that LEC projects directly to CA1 generated a model with poor fit. The depicted network model (LEC to septal CA3 to distal septal CA1 to proximal septal subiculum) also had good fit for group Sham Novel‐Novel (Fig. 7B). Consequently, when the data from the two conditions are collapsed and tested on the same network model, it still has acceptable fit (*χ* ^2^_9_ = 10.1, *P* = 0.35; CFI = 0.98; RMSEA = 0.07). When the same model was re‐tested for the Sham Novel‐Familiar group, but using proximal CA1 and distal subiculum (rather than distal CA1 and proximal subiculum) it had very poor fit. For the Novel‐Novel exploration condition, this variant (proximal CA1 and distal subiculum) retained acceptable fit.

Inspection of this model (see Fig. 7) suggested differences in the strength of some pathways in the Sham Novel‐Novel and Sham Novel‐Familiar groups. To test this formally, the data from the two groups were stacked (see Methods) onto the model in Figure 7. The resulting comparison revealed an overall difference between the groups (*χ* ^2^_6_ = 17.4, *P* = 0.008). Allowing the path between area 36 and area 35 to vary, significantly improved the fit of the model (*χ* ^2^_1_ = 5.11, *P* = 0.024), as did unconstraining the path between distal septal CA1 and the septal proximal subiculum (*χ* ^2^_1_ = 8.11, *P* = 0.004). There were no other path differences between the two behavioral conditions (path between area 36 and rostral LEC: *χ* ^2^_1_ = 3.13, *P* = 0.077; all other paths *χ* ^2^_1_ ≤2). The path differences between the two conditions were due to stronger effective connections in these two pathways (area 36 with area 35; distal septal CA1 → proximal septal subiculum) when the rats discriminate novel from familiar objects, rather than just explore novel objects (Fig. 7). These analyses reveal that the differences between the Novel‐Familiar and Novel‐Novel groups on this model are predominantly related to the strength of specific connections within the network, rather than the overall network structure.

Taken together, these analyses indicate that the overall network structure of parahippocampal‐hippocampal interactions was not altered by the behavioral condition. The best fitting networks were those that incorporated distal CA1 and proximal subiculum, i.e., those subareas most anatomically linked with perirhinal cortex and LEC. However, task demands appeared to alter the strength of specific pathways within this network; Novel‐Familiar discriminations were associated with greater linkage between areas 35 and 36, and from distal CA1 to proximal subiculum.

### Testing the Novel Object Network after Perirhinal Cortex Lesions

The novel object networks depicted in Figure 7 could not be tested with the two lesion groups, due to their lack of perirhinal tissue. Thus, their models began in caudal LEC (present in all four groups) while retaining the same hippocampal components. This alteration produced an acceptable simplex model that projected from cLEC to septal CA3, then to distal septal CA1, and finally to the proximal septal subiculum (Fig. 8). This model had good levels of fit for groups Perirhinal Novel‐Familiar discrimination (Fig. 8A), Perirhinal Novel‐Novel (Fig. 8C), and Sham Novel‐Novel (Fig. 8D). Group Sham Novel‐Familiar discrimination displayed a slightly elevated RMSEA, but all of the other indices indicated a well‐fitting model (Fig. 8B). The Fos activity in the regions included in the model for group Sham Novel‐Familiar discrimination are highly inter‐correlated, creating redundant information that can inflate the RMSEA (Tabachnick and Fidell, 1996).

The models for each group were then tested with the Fos counts from just Layers II, III, or V + VI of cLEC in place of the Fos counts from the whole cLEC. All other aspects of the network were held constant. Each of the three cortical layers fitted in place of the whole cLEC in groups Perirhinal Novel‐Familiar discrimination and Sham Novel‐Novel, while only cortical Layers II and III provide statistical fit in groups Sham Novel‐Familiar discrimination and Perirhinal Novel‐Novel (Table 1).

**Table 1:** Model Fit When Cortical Layers II, III or V+VI Replace Whole Caudal LEC Fos Counts in the Models Depicted in Figure 8

	***χ^2^‐value***	***df***	***P‐value***	***CFI***	***RMSEA***	***Acceptable model fit***
**Peri Novel‐Familiar**						
Cortical layer II	2.83	3	0.42	1.0	0.0	✓
Cortical layer III	2.46	3	0.48	1.0	0.0	✓
Cortical layers V+VI	1.58	3	0.66	1.0	0.0	✓
**Sham Novel‐Familiar**						
Cortical layer II	3.01	3	0.38	0.99	0.04	✓
Cortical layer III	1.72	3	0.63	1.0	0.0	✓
Cortical layers V+VI	9.75	3	0.021	0.83	0.45	x
**Peri Novel‐Novel**						
Cortical layer II	1.90	3	0.59	1.0	0.0	✓
Cortical layer III	0.50	3	0.92	1.0	0.0	✓
Cortical layers V+VI	4.14	3	0.25	0.49	0.22	x
**Sham Novel‐Novel**						
Cortical layer II	2.62	3	0.45	1.0	0.0	✓
Cortical layer III	2.31	3	0.51	1.0	0.0	✓
Cortical layers V+VI	0.7	3	0.88	1.0	0.0	✓

Statistical fit of structural equation models when lateral entorhinal cortex Layers II, III, or V + VI replace the counts for the whole caudal lateral entorhinal cortex in the models depicted in Figure 7. CFI, comparative fit index; df, degrees of freedom; Peri, perirhinal cortex lesion group; RMSEA, root mean square error of approximation.

The initial impression is that perirhinal cortex lesions had little impact on these parahippocampal‐hippocampal pathways (Fig. 8). The first test of this preliminary conclusion, involved stacking the data from all four groups on the network structure depicted in Figure 8. Allowing the coefficients of all paths to vary between all four groups did not significantly improve the fit over the constrained model (*χ* ^2^_9_ = 15.7, *P* = 0.074), indicating no overall difference between the four groups. Supporting this conclusion, the Fos data from all four groups were collapsed to create a single group on which the model could be tested. These combined data produced fit indices that just reached acceptable levels (*χ* ^2^_3_ =5.74, *P* = 0.13; CFI = 0.92; RMSEA = 0.14).

Next, the behavioral conditions were collapsed to focus on the effects of surgery, i.e., comparing between surgical status regardless of behavior. There was no difference between the constrained model and the model in which the weights of all paths were free to fluctuate (*χ* ^2^_3_ = 2.61, *P* = 0.46), implying no connectivity difference between the rats with perirhinal lesions and their surgical controls. To probe this null effect, group comparisons were made within the behavioral conditions. Again, there was no overall improvement of fit when the paths were allowed to vary as compared to when they were constrained to be the same between groups Sham Novel‐Novel and Perirhinal Novel‐Novel (*χ* ^2^_3_ = 3.19, *P* = 0.37). This was also the case when groups Sham Novel‐Familiar discrimination and Perirhinal Novel‐Familiar discrimination were compared (*χ* ^2^_3_= 5.66, *P* = 0.13). The inference is that perirhinal cortex lesions did not disrupt the patterns of hippocampal processing.

### Comparing Networks for Novel‐Novel Exploration vs. Novel‐Familiar Discrimination

The Fos data were next collapsed such that lesion status was ignored, so that all rats in the Novel‐Novel condition were compared with all rats in the Novel‐Familiar discrimination condition. Allowing the weights of the paths to be different yielded a model of significantly better fit than the constrained model (*χ* ^2^_3_ = 8.39, *P* = 0.039), indicating there was a network difference between the behavioral conditions (Figs. 8A,B vs. 8C,D). When paths were individually unconstrained, the only one to improve fit significantly was between distal septal CA1 and the proximal septal subiculum (*χ* ^2^_1_ = 7.35, *P* = 0.007). Subsequent pairwise stacking between the groups on this network model revealed a difference between groups Sham Novel‐Familiar discrimination and Sham Novel‐Novel (*χ* ^2^_3_ = 8.38, *P* = 0.039), which again was due to a difference in the path between distal septal CA1 and the septal proximal subiculum (*χ* ^2^_1_= 8.11, *P* = 0.004), but not in the other paths (*χ* ^2^_1_ ≤1). This path difference, based on behavioral condition, would appear to be largely driven by the surgical control animals as there was no overall difference when groups Perirhinal Novel‐Familiar and Perirhinal Novel‐Novel were stacked on the same model (*χ* ^2^_3_= 2.27, *P* = 0.52).

To summarize, stronger effective connections were observed between distal septal CA1 and proximal septal subiculum in the Novel‐Familiar discrimination condition than in the Novel‐Novel exploration condition. This difference was only seen, however, in the surgical control rats and not after perirhinal lesions. This difference suggests that the lesions attenuated the strength of this connection, however, as described in the previous section when groups Sham Novel‐Familiar discrimination and Perirhinal Novel‐Familiar discrimination were directly compared, there was no statistical difference, indicating that the lesions did not significantly affect this connection.

### Prelimbic Cortex and Nucleus Reuniens of the Thalamus—Sham Animals Only

Additional models of good fit were derived that involved prelimbic cortex (Fig. 9). These models were consistent with the finding that prelimbic cortex projects directly to the deep cortical layers of the perirhinal cortex and lateral entorhinal cortex, connections that are reciprocated (Conde et al., 1995; Vertes, 2004; Jones and Witter, 2007). Prelimbic cortex also projects indirectly to temporal CA1, via nucleus reuniens (Vertes et al., 2007; Prasad and Chudasama, 2013).

The first models were tested only in the surgical control groups, to retain the prelimbic–perirhinal link. The resulting optimal network models for these two sham groups (Figs. 9A,B) both involved a path between prelimbic cortex and nucleus reuniens, with a further connection with distal temporal CA1, while caudal LEC connected with the same CA1 region in both conditions. At the same time, there were group differences as the optimal model for the Sham Novel‐Familiar group involved a path between the prelimbic cortex and area 36 of the perirhinal cortex (Fig. 9A). In comparison, for the Sham Novel‐Novel group, prelimbic cortex was connected with area 35 of the perirhinal cortex and caudal LEC (Fig. 9B). There appeared to be differences between the behavioral conditions as testing the Fos data from the Sham Novel‐Familiar group on the optimal network for the Sham Novel‐Novel group (depicted in Fig. 9B) revealed a model of inadequate fit (*χ* ^2^_8_ = 13.9, *p* = 0.083 CFI = 0.92; RMSEA = 0.26). Likewise, poor indices of fit were generated when the data from group Sham Novel‐Novel were tested on the optimal model for Sham Novel‐Familiar discrimination (*χ* ^2^_9_ = 13.0, *P* = 0.16; CFI = 0.82; RMSEA = 0.20; model structure depicted in Fig. 9A).

These data suggest a functional connection between prelimbic cortex and perirhinal area 36 in the Novel‐Familiar discrimination condition, while during Novel‐Novel object exploration the prelimbic cortex is functionally connected with perirhinal area 35 and LEC (Figs. 9A,B). When the two groups were stacked on these models, no differences emerged (analyses not shown). Subsequently, the inter‐regional Fos correlations between prelimbic cortex and perirhinal cortex were compared directly between groups Sham Novel‐Familiar and Sham Novel‐Novel using Fisher's r‐to‐z transformation (Zar, 2010). The connection between prelimbic cortex and area 36 was significantly different between the two groups (*z* = 2.99, *P* = 0.003), with a stronger correlation in the Novel‐Familiar discrimination group (Fig. 9A).

### Prelimbic Cortex and Nucleus Reuniens of the Thalamus—All Four Groups

The final set of models was a subset of those described in the previous section but involved brain regions present in all four animal groups (Figs. 9C–F). The perirhinal cortices were, therefore, eliminated, creating a network model consisting of two parallel pathways between the prelimbic cortex and the distal region of temporal CA1; the first via nucleus reuniens and the second via caudal LEC (Figs. 9C–F). These networks generated models of good fit for both groups in the Novel–Novel objects condition (Figs. 9E,F). The same model had only poor fit for both groups in the Novel‐Familiar discrimination condition (Figs. 9C,D). In all four groups, the fit of the models did not depend on the direction of connections between nucleus reuniens, prelimbic cortex, and cLEC (Figs. 9C–F).

Additional evidence for behavioral condition differences emerged when the data from all four groups were collapsed to create a single group. These combined data were tested on the network structure depicted in Figures 9C–F, producing a model of poor fit (*χ* ^2^_2_ =6.71, *P* = 0.035; CFI = 0.92; RMSEA = 0.23), which indicated differences among the four datasets. However, when the weights of all of the paths in the model were constrained to have the same value for all four groups, the fit was no different from when the structural weights were free to differ between the groups (*χ* ^2^_12_= 5.49, *P* = 0.94). Subsequent pairwise stacking procedures yielded no differences in the same network (Figs. 9C–F) either by condition or by lesion (analyses not shown). Consequently, no individual group stood out as an outlier.

## DISCUSSION

Serial connections involving perirhinal cortex and lateral entorhinal cortex are assumed to convey object‐related information for hippocampal processing (Bussey et al., 2007; Diana et al., 2007; Barker and Warburton, 2011b; Knierim et al., 2014). Support comes from rat studies showing that while perirhinal cortex lesions impair object recognition (Mumby and Pinel, 1994; Brown and Aggleton, 2001), lesions of the lateral entorhinal cortex and hippocampus can impair object‐context recognition, object‐in‐place recognition, and object recency (Winters et al., 2004; Jo and Lee, 2010; Warburton and Brown, 2010; Barker and Warburton, 2011b; Albasser et al., 2012; Wilson et al., 2013a, 2013b). Further, both perirhinal and lateral entorhinal units respond to objects while lateral entorhinal units can also secondarily represent spatial information (Deshmukh and Knierim, 2011; Deshmukh et al., 2012). Consequently, the lateral entorhinal cortex could help place objects in the context of other local/proximal cues (Neunuebel et al., 2013). Thus, while perirhinal cortex is sufficient to signal object familiarity (Ho et al., 2015), interactions with the hippocampus, via entorhinal cortex, are required for object‐based associative learning. The present study examined this information transfer using a combination of lesions and c‐*fos* imaging. The results not only reveal that the hippocampus and perirhinal cortex can function independently but also provide converging evidence for preserved signals of object novelty in the absence of perirhinal cortex. Evidence was also found reinforcing the notion that the proximal‐distal axis within hippocampal subfields contains changing functions.

The behavioral findings showed that although the perirhinal lesions impaired, as expected, the preference for novel over familiar objects when both are presented together (Novel‐Familiar), overall levels of object exploration appeared unaffected. This null result is informative as it shows that the c‐*fos* comparisons were not confounded by exploration levels. Equally informative was the increased exploration by rats with perirhinal lesions seen during the Novel‐Novel condition compared to the Novel‐Familiar condition, which was equivalent to that in the sham controls. Thus, perirhinal lesions did not cause novel objects to seem familiar. This preserved sensitivity to the presence of novelty corresponds to the frequent finding that rats with perirhinal lesions show normal levels of ‘sample phase’ exploration (Winters et al., 2004; Barker et al., 2007; Bartko et al., 2007a, 2007b; Mumby et al., 2007; Albasser et al., 2009; McTighe et al., 2010; Barker and Warburton 2011a; Albasser et al., 2015; Olarte‐Sanchez et al., 2015). These findings correspond because during the ‘sample phase’ of any spontaneous recognition memory test, rats explore a novel object without the presence of a familiar object. This same sensitivity to the presence of novelty does, however, contrast with the impaired discrimination performance in the Novel‐Familiar condition and with prior evidence that perirhinal lesions can bias novel stimuli to seem familiar (McTighe et al., 2010; Romberg et al., 2012). This lesion‐induced bias was thought to arise from an increased sensitivity to proactive interference (Cowell et al., 2010; McTighe et al., 2010). This interference account appears inconsistent, however, with the lack of a perirhinal lesion effect on exploration levels across blocks of trials when given just novel objects (Fig. 3F) or for the lack of a reduction in overall exploration across blocks of trials for the Novel‐Familiar condition.

Instead, the present behavioral results reveal that rats with perirhinal cortex lesions can still detect the presence of a novel object (Novel‐Novel), but struggle to discriminate its identity (Novel‐Familiar) (see also Albasser et al., 2015; Olarte‐Sanchez et al., 2015). Implicit within this conclusion is the assumption that other brain sites can detect novelty in the absence of the perirhinal cortex, even if that novelty information may not guide recognition discriminations. This conclusion is reinforced by the present Fos results; perirhinal lesions scarcely affected the patterns of Fos‐related activity seen in other brain sites responsive to novel stimuli, including the hippocampus. Instead, the impact of perirhinal lesions was restricted to the subiculum and layer III of entorhinal cortex, sites that receive direct inputs from perirhinal cortex (Burwell and Amaral, 1998; Furtak et al., 2007). In the hippocampus, interactions as measured by patterns of Fos production, appeared insensitive to the loss of perirhinal cortex. This apparent structural independence complements an earlier study showing that hippocampal lesions spare parahippocampal c‐*fos* interactions (Kinnavane et al., 2014).

The degree of independence of the hippocampus from perirhinal cortex is particularly noteworthy as novel stimuli typically evoke a characteristic pattern of interlinked IEG activity in intact rats: perirhinal cortex → lateral entorhinal cortex → dentate gyrus/CA3 → CA1 (Poirier et al., 2008; Albasser et al., 2010; Kinnavane et al., 2014). In contrast, familiar stimuli are associated with interlinked cortical activity that reaches CA1 more directly: perirhinal cortex → lateral entorhinal cortex → CA1 (Poirier et al., 2008; Albasser et al., 2010; Olarte‐Sánchez et al., 2014; Kinnavane et al., 2014). The most parsimonious account is that familiarity is the absence of novelty (or vice versa). The loss of perirhinal cortex, with the concomitant loss of novelty signals (Ho et al., 2015) might, therefore, have been expected to bias entorhinal activity towards the temporoammonic CA1 (familiarity) pathway. The failure to find evidence of a switch to this more direct pathway after perirhinal lesions in both the Novel‐Novel and Novel‐Familiar conditions strongly suggests that extra‐perirhinal sites can still signal the presence of novel objects to the hippocampus, but cannot attribute this information to the relevant stimulus.

Additional evidence for the existence of extra‐perirhinal novelty/familiarity signals comes from habituation studies, where perirhinal lesions do not block the reduction in reactivity to repeated stimuli (Amin et al., 2006; Mumby et al., 2007; Robinson et al., 2009; Albasser et al., 2011b, 2009). Possible sources of this spared (non‐perirhinal) information, which reaches the rat lateral entorhinal cortex, include visual association area Te2 (Ho et al., 2011), the piriform, postrhinal, insular, and frontal cortices (Kerr et al., 2007). In addition, amygdala lesions were found to selectively impair familiarity‐based behavior in the rat, while not altering overall recognition memory (Farovik et al., 2012). Another potential source comes from dopaminergic signals, which have been linked to changes in hippocampal activity depending on novelty (Lisman and Grace, 2005; Lisman et al., 2011).

This study also refined network models of medial temporal IEG activity. Optimal SEM networks matched known proximal‐distal connectivity patterns within CA1 and the subiculum. Thus, the results support the concept of greater object‐based processing in distal CA1 and proximal subiculum, which contrasts with more spatial‐based processing in proximal CA1 and distal subiculum (Aggleton, 2012; Ranganath and Ritchey, 2012; Nakamura et al., 2013; Knierim et al., 2014). A further goal was to compare entorhinal laminae, given their different hippocampal targets (Fig. 1). Fos levels in both layers II and III, i.e., hippocampal afferent layers, fitted all acceptable hippocampal input models (Table 1). There was, however, a high level of covariance between these laminae, both of which receive perirhinal inputs (Burwell and Amaral, 1998). Meanwhile, Fos levels within the deep layers of entorhinal cortex, i.e., the hippocampal output layers, only inconsistently fitted these same models (Table 1). This pattern of results only partially matches the initial prediction that perirhinal novelty signals bias processing towards entorhinal layer II (and then to dentate/CA3). Instead, performing recognition discriminations (Novel‐Familiar) led to higher Fos counts in both layers II and III of LEC (but not V‐VI) than when experiencing just novelty (Novel‐Novel). In this respect, one potentially important factor is how prefrontal areas might affect medial temporal activity (Sigurdsson and Duvurci, 2016).

In the present experiment, absolute Fos‐counts in CA1 rather than CA3 were found to be sensitive to the quantity of novel objects. This result may seem counterintuitive as IEG network analyses (described above) particularly associate CA3 with novel object processing (Albasser et al., 2010; Olarte‐Sánchez et al., 2014; Kinnavane et al., 2014). It is, however, the case that CA1 is involved in all of these IEG network models of object processing (novel and familiar). Additionally, both cortical layers II or III of LEC (hippocampal afferent regions) consistently produce models of good fit for novel object processing (Kinnavane et al., 2014; present data). Taken together these data indicate that rather than object class generating a clear dichotomy between laminar routes into the hippocampus, information relating to novel stimuli could access the hippocampus via both the perforant and temporoammonic pathways, while familiar information is more confined to the temporoammonic pathway. This notion echoes a mechanism recently demonstrated in an electrophysiological study in rats. It was found that simultaneous activation of CA1 pyramidal neurons by inputs originating in both CA3 and cortical layer III of the entorhinal cortex was necessary and sufficient to induce the formation of new place fields and contextual feature selectivity (Bittner et al., 2015).

An unexpected, but striking, difference between the Sham Novel‐Familiar and Sham Novel‐Novel groups was that the former animals had over five times the number of significant inter‐area correlations (320/465) than the Novel‐Novel animals (63/465). Discriminating novel from familiar objects was associated, for example, with higher effective connectivity between areas 36 and 35 of the perirhinal cortex (Fig. 7), and between CA1 and the subiculum (Figs. 8B,D). This contrast suggests possible top‐down influences based on the differing task demands. Of particular relevance, therefore, were the stronger prelimbic – perirhinal Fos links for the Novel‐Familiar condition than the Novel‐Novel condition (Figs. 9A,B). Although prelimbic cortex is not itself required for simple recognition memory tasks (Barker et al., 2007; Cross et al., 2013), its executive functions may include guiding patterns of object exploration based on previously acquired contextual information (Preston and Eichenbaum, 2013). The present analyses suggest a form of executive control that helps to synchronize activity across constituent sites. These same analyses also support the notion that nucleus reuniens provides a key link between prelimbic cortex and CA1 (Prasad and Chudasama, 2013; Sigurdsson and Duvarci, 2016).

The c‐*fos* analyses took place 8‐9 months post‐lesion, in order to look at well‐established behavioral and activity consequences. The rationale follows from the study of patients with medial temporal lobe damage, who show stable memory deficits many years after the initial insult (Baddeley et al., 2001; Corkin, 2002; Mayes et al., 2004; Aggleton et al., 2005; Barbeau et al., 2005; Bowles et al., 2010; Dede et al., 2013). While it remains possible that the lengthy survival time in the present study may have promoted some compensation processes, other behavioral studies of perirhinal lesions report persistent deficits in object recognition (Albasser et al., 2015, 2013). Furthermore, although the effects of different survival times on c‐*fos* expression after perirhinal lesions have not been examined, it is known that distal IEG changes following anterior thalamic lesions do not diminish with time after surgery (Poirier and Aggleton, 2009).

The current study was prompted by neuropsychological evidence of double dissociations in function between the perirhinal cortex and hippocampus, despite their many interconnections (Graham and Hodges, 1997; Winters et al., 2004; Bowles et al., 2007, 2010, 2011). The present findings began by complementing those of a study into the impact of hippocampal lesions on perirhinal c‐*fos* (Kinnavane et al., 2014), where the preservation of parahippocampal Fos activity was the dominant result. These same sets of IEG findings are echoed by behavioral studies of rats. For example, configural discriminations that rely on learning associations between elements in a visual stimulus are sensitive to hippocampal, but not perirhinal, lesions (Sanderson et al., 2006; Aggleton et al., 2010), contrasting with the latter region's relatively greater importance for object recognition (Mumby et al., 2001; Winters et al., 2004). The present results show a need to move to a more anatomically rich concept of perirhinal–medial temporal interactions, which are not simply a serial progression that lead to the hippocampus at the apex. This need is highlighted by the apparent presence of multiple sources of object‐based information, only some of which enable object recognition when novel and familiar objects are presented simultaneously (Albasser et al., 2015). Consequently, the findings support a call to avoid the simple dichotomy between “what” and “where” pathways in the medial temporal lobe (Knierim et al., 2014).
